# Safety of trastuzumab (Herceptin^®^) during pregnancy: two case reports

**DOI:** 10.1186/1757-1626-2-9329

**Published:** 2009-12-16

**Authors:** Matthew J Goodyer, Jeffri RM Ismail, Seamus P O'Reilly, Eugene J Moylan, C Anthony M Ryan, Paul AF Hughes, Alan O'Connor

**Affiliations:** 1 Department of Medical Oncology, Cork University Hospital, Cork, Ireland; 2 Department of Medical Oncology, Kerry General Hospital, Tralee, Ireland; 3 Department of Paediatrics, Cork University Hospital, Cork Ireland; 4 Department of Obstetrics and Gynaecology, Kerry General Hospital, Tralee, Ireland

## Abstract

We report on two cases of women on trastuzumab therapy for breast cancer who became pregnant and delivered healthy live infants. At the time of reporting the children are growing and developing normally (ages 3 and 2).

## Background

Trastuzumab (Herceptin^®^) is a monoclonal IgG1 antibody to the human epidermal growth factor HER2/neu receptor which is indicated in the treatment of breast cancers over-expressing the HER2 receptor. Although in preclinical studies placental transfer occurred in monkeys, trastuzumab has not been directly shown to cross the human placenta. However placental transfer of IgG1 is documented [1] and has been shown for other IgG1 antibodies rituximab[2] and infliximab[3]. There is little information in the literature regarding the safe use of trastuzumab in pregnancy, although some reports suggest an increased risk of oligohydramnios during the second and third trimesters[4,5], and the drug carries a United States Food and Drug Administration category D warning label (there is positive evidence of human fetal risk, but the benefits from use in pregnant women may be acceptable despite the risk) [6].

## Case Presentation 1

A 30 year old female was diagnosed with a locally advanced right breast cancer for which she received neoadjuvant chemotherapy with doxorubicin and cyclophosphamide followed by docetaxel. She then proceeded to mastectomy and axillary node clearance for node positive, hormone receptor negative disease, followed by adjuvant radiotherapy. Subsequently, she underwent left mastectomy.

She conceived whilst clinically disease-free and had an uneventful first trimester. During the second trimester she presented with right-sided rib pain and a malignant pleural effusion. HER2/neu receptor studies on the primary tumor at the time of relapse were positive, and she was commenced on single agent trastuzumab.

At 29 weeks gestation, she gave birth to a live female weighing 1220 g by elective caesarean section. She did not breast-feed. The infant's postnatal period was complicated by respiratory distress syndrome, and concern about conductive hearing loss, which subsequently resolved.

During ongoing paediatric follow-up, the baby was noted to have mild hypertonia and hyperreflexia, which subsequently resolved, and minimal tightening of her left Achilles tendon. At the current age of 3, the child is cognitively normal with height at the 50th percentile, and weight and head circumference at the 25th centile. She has ongoing minimal tightness of her left Achilles tendon, but no obvious neurological deficit.

## Case Presentation 2

A 36 year old female was diagnosed with locally advanced cancer of the right breast, for which she underwent total mastectomy and axillary clearance. The tumour was hormone receptor negative and HER2 receptor positive.

She received adjuvant doxorubicin and cyclophosphamide followed by docetaxel, as well as chest wall and regional radiation.

She was subsequently enrolled in the Herceptin^® ^Adjuvant (HERA) trial, which compared adjuvant trastuzumab to observation in women with HER2-positive primary breast cancer who had completed adjuvant chemotherapy [7]. She was initially randomised to the observation control arm, but was later offered trastuzumab treatment after the preliminary results of the HERA study were reported (as permitted by a change in the study protocol).

The patient was found to be 6 weeks pregnant, at which stage she had received 6 doses of trastuzumab. No further trastuzumab was administered. The patient had had 3 previous pregnancies, 2 of which had resulted in spontaneous abortions.

Two gestational sacs were shown on initial ultrasound imaging, with only one sac showing a live fetus, a finding confirmed on subsequent scans. The patient elected to continue the pregnancy which was otherwise uncomplicated. At 39 weeks gestation a healthy female infant (birth weight 2.94 kg) was born by normal vaginal delivery.

The baby was formula fed. The infant was admitted for gastroenteritis at the age of 3, 8 and 11 months of age, but recovered well from each episode. Subsequent growth and development have been normal with the child now aged 2.

## Discussion

Breast cancer is relatively uncommon in pregnancy, with up to 1/3000 live births affected. Cancers tend to be more aggressive and advanced at presentation, with a resultant poorer prognosis. They are also often associated with other poor prognostic factors such as lymphovascular invasion, hormone insensitivity, and over-expression of HER2/neu. As a class of chemotherapeutic drugs, anthracyclines have the largest safety data in pregnancy, with a low rate of congenital malformations, mostly occurring in the setting of first-trimester exposure [8].

Only 8 case reports currently exist in the literature with regards to the use of trastuzumab in pregnancy (Table 1). Watson described a case of anhydramnios occurring at 23 weeks' gestation during adjuvant trastuzumab treatment commenced pre-conception. This resolved slowly after drug cessation [5].

**Table 1:** Trastuzumab use during pregnancy

	Case 1	Case 2	Watson^**5**^	Fanale^**9**^	Waterston^**10**^	Bader^**4**^	Shrim^**11**^	Sekar^**12**^	Witzel^**13**^	Pant^**14**^
Maternal age	33	38	28	29	30	38	32	28	~32	~33

Stage	IV	III	II	IV	II	IV	IV	IV	IV	IV

Treatment	T	T	T	T/V	T	T/P	T	T/D	T	T

Initiation	2^nd ^trimester	Pre	Pre	27/40	Pre	25/40	Pre	23/40	Pre	Pre

Completion	29/40	6/40	20/40	34/40	1/40	28/40	24/40	27/40	NS	30/40

Antenatal complications	None	1 of 2 viable fetal sacs	None	Oligo	None	Anhydramnios "Renal failure"	None	Anhydramnios	Oligo, Vaginal Bleeding	Oligo

Delivery	CS	VD	VD	VD	VD	CS	CS	CS	CS	VD

Gestation	29	39	37.5	34+5	Term	32+1	37	36+2	28	32+1

Perinatal Comp.	Nil	Nil	Nil	Nil	Nil	Fetal distress	Breech	Breech	Bleeding	Nil

Sex	F	F	F	M	F	M	F	M	F	F

Birthweight	1220 g	2940 g	2960 g	2270 g	NS	1460 g	2600 g	2230 g	1015 g	1810 g

Neonatal	RDS	Normal	Normal	Normal	NS	Sepsis, RDS	RDS	Normal	Multiorgan Failure	Normal

Outcome	Nil significant	Normal	Normal	Normal	NS	Normal	Normal	Normal	Neonatal death	Normal

Duration of follow-up	3 years	2 years	6/12	6/12	NS	3/12	2/12	NS	21 weeks	60/12

T = Trastuzumab, V = Vinorelbine, P = Paclitaxel, D = Docetaxel, Pre = Pre-pregnancy, NS = Not stated, Oligo = Oligohydramnios, CS = Caesarean section, VD = Vaginal Delivery, RDS = Respiratory Distress Syndrome

Fanale et al reported on a patient with metastatic breast cancer treated with combination vinorelbine and trastuzumab, with good disease response, and a healthy male infant born at 34 weeks gestation [9].

Waterston and Graham described a patient receiving adjuvant trastuzumab, who conceived while on treatment. At the time of confirmation of pregnancy in the first trimester, the patient had already received 3 cycles of trastuzumab. The drug was stopped, and a normal pregnancy and birth ensured, with a healthy female delivered and no long-term sequelae reported [10].

Bader et al reported on a patient treated at 26 and 29 weeks gestation with trastuzumab and paclitaxel for metastatic breast cancer presenting during pregnancy. The fetus developed anhydramnios due to renal failure. A male infant was born at 32 weeks by elective caesarean section. Serum creatinine was mildly elevated and ultrasound of kidneys showed transient hyperechodensities, but these abnormalities resolved, and a healthy infant was discharged at age 6 weeks. Development was normal at 12 weeks [4].

Shrim et al described a patient on maintenance single-agent trastuzumab for metastatic breast cancer who unexpectedly conceived. Trastuzumab was continued until 24 weeks gestation, when it was stopped for asymptomatic persistent mildly reduced ejection fraction. The pregnancy was otherwise uneventful. A healthy female was born at 37 weeks gestation by caesarean section (performed due to breech presentation), with ensuing normal growth and development noted in the short-term [11].

Sekar and Stone reported on a lady who presented at 20 weeks gestation with metastatic breast cancer and received docetaxel and trastuzumab. An ultrasound 4 weeks after 2 cycles of chemotherapy revealed anhydramnios. A follow-up ultrasound 7 weeks post chemotherapy showed reappearance of amniotic fluid [12].

Witzel and Müller described a patient with metastatic breast cancer, treated with trastuzumab and vinorelbine with disease remission followed by maintenance trastuzumab. The woman conceived while on maintenance therapy. At 27 weeks gestation, the patient presented with oligohydramnios and vaginal bleeding. Caesarean section was subsequently performed for ongoing bleeding. The newborn infant developed respiratory failure, capillary leak syndrome, persisting infections and necrotizing enterocolitis, and ultimately died [13].

Finally, Pant et al reported on a patient treated with paclitaxel and trastuzumab for metastatic breast cancer with excellent disease response, and subsequent maintenance trastuzumab. She was unexpectedly found to be pregnant at 14 weeks gestation, but elected to continue receiving treatment. After several weeks of low normal amniotic fluid volume, the patient developed oligohydramnios at 32 weeks gestation and a caesarean section was performed. A healthy child was born with no immediate or longer term (up to 5 years of age) sequelae noted [14].

A mechanism proposed for the association between trastuzumab use in the expectant mother and the development of oligohydramnios is that epidermal growth factor receptors are expressed in the fetal kidney during development, inducing DNA synthesis and mitosis, and that blocking these receptors, such as might occur with trastuzumab, leads to a decrease in kidney cell proliferation [1].

## Conclusion

Although information regarding the safety of trastuzumab for breast cancer during pregnancy is scant, there appears to be an increased incidence of oligohydramnios associated with the use of the agent. This was reversible on discontinuation of the drug in most cases. Trastuzumab was associated with neonatal death in one instance. Despite this, the majority of infants did not appear to have suffered significant long-term sequelae resulting from the use of the agent and its benefit in disease control warrants consideration of use during pregnancy (particularly beyond the first trimester) with careful monitoring of fetal well-being (including amniotic fluid volume).

## Patient's Perspective

"My daughter is now 3 1/2 years and is perfectly normal. I would have no problem recommending Herceptin during pregnancy. If any concerned patient would like to contact me and see my daughter I would be glad to put their mind at ease."

## Consent

Written informed consent was obtained from the patient's next-of-kin, who also gave consent for their child (Case Presentation 1) and from the patient, who also gave consent for her child (Case Presentation 2) for publication of this case presentation. A copy of the written consent is available for review from the journal's Editor-in-Chief.

## Competing interests

The authors declare that they have no competing interests.

## Authors' contributions

MJ Goodyer wrote up Case Presentation 1, performed the literature search, and compiled all other sections, with the exception of Case Presentation 2.

JRM Ismail wrote up Case Presentation 2.

SP O'Reilly and EJ Moylan, Medical Oncologists, CAM Ryan, Paediatrician, and PAF Hughes, Obstetrician and Gynaecologist provided consultant oversight and made several recommendations on earlier drafts which were incorporated into the final version.

A O'Connor, as surviving spouse of the patient in Case Presentation 1, provided a patient's perspective, for which he is happy to be acknowledged.

All authors read and approved the final manuscript.
